# The Chick Embryo Chorioallantoic Membrane as an *In Vivo* Assay to Study Antiangiogenesis

**DOI:** 10.3390/ph3030482

**Published:** 2010-03-08

**Authors:** Domenico Ribatti

**Affiliations:** Department of Human Anatomy and Histology, University of Bari Medical School, Piazza G. Cesare, 11, Policlinico 70124, Bari, Italy; Email: ribatti@anatomia.uniba.it; Tel.: +39-080-547-8240; Fax: +39-080-547-8310.

**Keywords:** antiangiogenesis, chorioallantoic membrane, tumor progression

## Abstract

Antiangiogenesis, e.g., inhibition of blood vessel growth, is being investigated as a way to prevent the growth of tumors and other angiogenesis-dependent diseases. Pharmacological inhibition interferes with the angiogenic cascade or the immature neovasculature with synthetic or semi-synthetic substances, endogenous inhibitors or biological antagonists. The chick embryo chorioallantoic membrane (CAM) is an extraembryonic membrane, which serves as a gas exchange surface and its function is supported by a dense capillary network. Because its extensive vascularization and easy accessibility, CAM has been used to study morphofunctional aspects of the angiogenesis process *in vivo* and to study the efficacy and mechanism of action of pro- and anti-angiogenic molecules. The fields of application of CAM in the study of antiangiogenesis, including our personal experience, are illustrated in this review article.

## 1. Introduction

Angiogenesis depends on the balance of several stimulating and inhibiting factors [1]. Antiangiogenesis as a way of treating primary tumors and reducing their metastases, was first proposed by Judah Folkman in 1971 [2]. Beginning in the 1980s, the biopharmaceutical industry began exploiting the field of antiangiogenesis for creating new therapeutic compounds for modulating new blood vessel growth in angiogenesis-dependent diseases.

Antiangiogenic approaches fell in two categories: (a) agents that blocked the activity of pro-angiogenic molecules; (b) agents that directly affected endothelial cell function or survival (Table 1). 

**Table 1:** Direct and indirect angiogenesis inhibitors.

Direct	Indirect
Angiostatin; Bevacizumab (Avastin) Arresten; Canstatin; Combrestatin Endostatin; Thrombospondin Tumstatin; methoxyestradiol; Vitaxin	Targeting EGF-receptor tyrosine kinase Targeting VEGF receptor Targeting PDGF receptor Targeting PDGF receptor Targeting ERBB-2 Targeting interferon alpha receptor*

* Interferon alpha can be considered both a direct angiogenesis inhibitor, because it inhibits endothelial-cell migration, and an indirect angiogenesis inhibitor because it inhibits synthesis of FGF-2 by tumor cells.

Endogenous inhibitors of angiogenesis are defined as proteins or fragments of proteins that are formed in the body and can inhibit the formation of blood vessels (Table 2) [3]. 

**Table 2:** Endogenous inhibitors of angiogenesis.

Matrix derived	Growth factors and cytokines
Arresten Canstatin Endorepellin Endostatin Fibronectin fragment (Anastellin) Targeting fibronectin-binding integrins Fibulin Thrombospondin-1 and –2 Tumstatin	Interferons Interleukins Pigment epithelium derived factor (PEDF)
**Fragments of blood coagulation factors**	**Others**
Angiostatin Antithrombin-III Prothrombin kringle 2 Platelet factor-4	Tissue inhibitors of metalloproteinases (TIMPs) Chondromodulin 2-methoxyestradiol Prolactin fragments PEX Soluble Fms-like tyrosine kinase-1 (S-Flt-1) Troponin I Vasostatin

At least 27 different proteins and small molecules exist in the body whose function is to act as inhibitors of angiogenesis. These angiogenesis inhibitors can be detected in blood circulation, suggesting that they function in the angiogenic balance as endogenous angiostatic regulators even under physiological conditions [3].

Today, over 20 angiogenic growth factors and over 300 antiangiogenic molecules targeting different signalling pathways are being tested for their anticancer properties at preclinical and clinical stages. Although the results of the clinical trials are encouraging, the effects are modest. Clinical practice reveals that therapy with angiogenesis inhibitors does not prolong survival of cancer patients for more than months, because tumors elicit resistance.

Owing to its central role in promoting tumor growth, vascular endothelial growth factor (VEGF) has become a key therapeutic target and its function can be blocked at different levels of the signalling pathways. The majority of Food and Drug Adminstration (FDA)-approved angiogenesis inhibitors, as well as those in phase III clinical trials, neutralize VEGF, target its receptors or suppress its expression by tumor cells. Avastin (bevacizumab), a humanized anti-VEGF monoclonal antibody, has been the first angiogenesis inhibitor that was tested in multi-center clinical trials against cancer. In 2004, it was approved by the FDA as a first-line treatment for metastatic clorectal cancer in combination with 5-fluorouracil-based chemotherapy regimens [4].

## 2. The Chorioallantoic Membrane in the Study of Antiangiogenesis

The classical assays for studying angiogenesis *in vivo* include the rabbit ear chamber, the mouse dorsal skin and air sac, the chick embryo chorioallantoic membrane (CAM), the iris and avascular cornea of the rodent eye and the zebrafish [5]. 

*In vivo* angiogenesis assays have allowed important progress in elucidating the mechanism of action of several angiogenic factors and inhibitors. The main determinants dictating the choice of method are their cost, ease of use, reproducibility, and reliability. However, *in vivo* angiogenesis assays may be very sensitive to environmental factors and not readily accessible to biochemical analysis. Also, their interpretation is frequently complicated by the fact that the experimental condition adopted may inadvertently favour inflammation. In this case the angiogenic response is elicited indirectly, at least in part, through the activation of inflammatory or other non-endothelial cell types. 

The CAM is an extraembryonic membrane formed on day 4 of incubation by fusion of the chorion and the allantois. Immature blood vessels, lacking a complete basal lamina and smooth muscle cells, scattered in the mesoderm grow very rapidly until day 8 and give rise to a capillary plexus, which comes to be intimately associated with the overlying chorionic epithelial cells and mediates gas exchange with the outer environment. At day 14, the capillary plexus is located at the surface of the ectoderm adjacent to the shell membrane. Rapid capillary proliferation continues until day 11; thereafter, the endothelial cell mitotic index declines rapidly, and the vascular system attains its final arrangement on day 18, just before hatching [6].

CAMs are cultured either *in ovo* or *ex-ovo* as a shell-less culture in Petri dishes and plastic wrap/cup apparatus. There is no clear evidence that there is any significant difference between data derived using *in ovo* or shell-less culture method. It has been demonstrated that survival rate of eggs cultured *ex ovo* is the major success limiting step in this culture technique [6].

Focal application of test and control substances is still the most used method. It is quick and semi-quantifiable, economical, good for the screening of many novel substances. The one limitation of this approach concerns quantification of interaction of antiangiogenic drugs with CAM vessels rather than with pro-angiogenic molecules [6]. 

There are a variety of application methods or carriers described in literature to test angiogenic or antiangiogenic activity. The test material is usually introduced in the form of small filter disks, or small pieces of polymerized materials, such as gelatin sponges or biologically inert synthetic polymers. Blood vessels can be analyzed in terms of the number, diameter, density, permeability, branch point number and blood flow [6].

We have developed a new method for the quantitation of angiogenesis and antiangiogenesis in the CAM. Gelatin sponges treated with a stimulator or an inhibitor of blood vessel formation are implanted on growing CAM on day 8 [7]. Blood vessels growing vertically into the sponge and at the boundary between sponge and surrounding mesenchyme, are counted morphometrically on day 12. The newly formed blood vessels grow perpendicularly to the plane of the CAM inside the sponge, which does not contain preexisting vessels and can be quantified by morphometric evaluation of histologic CAM sections. More sophisticated techniques have been designed recently to perform reliable quantitative evaluation of vascular density, including *in ovo* cell proliferation, layered expression scanning to visualize the protein of interest, and fluorescent confocal microscopy of new blood vessels formation at the site of application.

The development of an avascular zone or a zone of inhibition at the site of application is considered indicative of antiangiogenesis. It was initially described by Taylor and Folkman [8] who showed that protamine produced an avascular zone when applied to the leading edge of the CAM. In studies of inhibition of angiogenesis (Table 3), there are two approaches which differs in the target vessels, i.e. those which examine the response in the rapid growing CAM blood vessels and those that evaluate the inhibition of angiogenic response induced by a well known angiogenic cytokine, usually fibroblast growth factor-2 (FGF-2) or VEGF.

**Table 3:** Testing Antiangiogenic Substances in the CAM Assay.^*^

AAV-mediated gene transfer of TIMP-1 [9]; AA98V (H)/L [10]; A-beta peptides [11]; Aeroplysinin-1[12]; Adiponectin [13]; Ad-vasostatin[14]; Agkistin [15]; AGM-1470 [16]; Alliin [17]; a4-b1antagonists[18]; Av-b3/av-b5 antagonists and ab [19,20,30,84,85,165,313]; Amifostine [21]; Amiloride [22]; Aminopeptidase-N antagonists [23]; Angioinhibins [24]; Ang-2 [25]; Angiostatin [26]; Angiotensinogen [27,28]; Anthracyclines and titanocene dichloride [29]; Antibacterial substances [31]; Antibiotics [32]; Ab anti-FGF-2 and anti-VEGF [33,34]; Anti-CD146 Mab [35]; Anti-collagen IV ab [36]; Antioxidant molecules [37]; Antithrombin [38]; Apicidin [39]; Aplidine [40]; Apolipoprotein(a) kringle V [41]; Apomorphine [42]; AQP-1 siRNA [43]; Arginine deaminase [44]; 2-aroylindoles [45]; Arresten [46]; Artesunate [47]; Ascorbic acid [48]; Atiprimod [49]; Aurintricarboxylic acid [50]; Azaspirine [51]
Bactericidal/permeability-increasing protein [52]; Bacterium PB[53]; Baicalein/baicalin [54]; Bleomycin [55]; Blockers of volume-regulated anion channels [56]; Beta-cyclodextrintetradecasulfate [57]; Beta-Escin [58]; Beta-HISV[59]; BMP-9 [60]; Bortezomib [61]; Butyric acid [62]
CAI [63]; Campesterol [64]; Canstatin [65]; Capsaicin [66]; Carbon materials [67]; Carrageenan [68]; Cartilage [69]; Catechins [70]; Cerivastatin [71]; Cheiradone [72]; Chemokine antagonist M3 [74]; Chondrocyte derived inhibitor [69]; Chondromodulin-1 [75]; Chrysin [76]; Cigarette smoke condensate [77,191]; Clodronate [78]; Clotrimazole [79]; Contortrostatin [80]; Curcumin [81,82]; COX inhibitors [86,87]; Cyclopeptidic VEGF inhibitor [88]; Cyclosporin [89]; Cytocholasin D [90]
7-Deazaxanthine [91]; Deguelin [92]; Delphinidin [93]; Deoxycholic acid-heparin conjugate [94]; Deoxycytidine nucleoside [95]; DFMO α-difluoromethylornithine [96]; Diaminoanthraquinone [97]; Dichloropy ridodithienenotriazine [98]; Dihydroartemisinin [99]; Dihydrotanshinone I [100]; Digoxin [101]; Ditriazine derivative [102]; DPTH-N10 [103]; Docetaxel [104]; Dominant-negative p65 PAK peptide [105]; Doxazosin [106]; Doxycycline [107]; Doxorubicin [108,209]
Eclipta prostata [109]; Emodin [110]; Endocannabinoid anandamide[111]; Endorepellin [112]; Endostatin [113,114,242]; Enoic acanthonic acid [86]; Eponeomycin [115]; Epoxyeicosatrienoic acid antagonist [116]; Escherichia Coli K5 polysaccharide derivatives [73,299]; Estrogen antagonists [117]; Ets-1 antisense [118,119]; Evodiamine [120]
Fascaplysin [121]; Fenretinide [122]; Flavonoids [123,261]; Fluorosynerazols [124]; Fractalkine [125]; Gangliosides [126]; Gastrodia elata [127]; Genepin [128]; Ghrelin [129] Gleditsia sinesis [130]; Glycine [131]; Goniodomin A [132] Grateloupia longifolia polysaccharide [133]; Green tea [134] ; Grifola frondosa [135]; GRO-beta [136]; GW654652[137]
Heparan sulfate suleparoide [138]; Heparanase [139]; Heparin or heparin fragments+cortisone [57,140,141,142,285]; HGF-like basic hexapeptides [143]; Herbamycin [144]; Histidine-proline-rich glycoprotein [145]; HIV-1 protease inhibitors [146]; Hox D10 [147]; Homocysteine [148]; HST-1 protein [149]; Human neutrophil peptides [151]; Hydroxycamptothecin [152]; Hyperforin [154]; Hypertermia [155]; Hypoestoxide [156]; Hypoxic eytotoxin TX-402 [157]; Hypoxia cytotoxins [158]
Indinavir and saquinavir [146]; Indolin-2-ketone compound [159]; Inhibitors of basement membrane biosynthesis [160,161,162]; Inhibitors of DNA methyltransferase [163]; IGF binding protein [164]; IL-12, -18, -21, 27 [166,167,168,169]; Ionizing radiation [170]; Isoflavones [171]; Isoprostanes [172]; Isosorbide mono-dinitrate [173]; JNI-17029259 [174]; JNI-26076713 [175]
KIN-841 [176]; Kininogen, kininogen-derived polyptides, kinostatin and related Mab [177,178,179,200]; KV11 [180]; Lactacystin [181]; Lambda-carragenan oligosaccharide [68]; Laminin-derived peptide [182]; Lamininarin sulphate [183]; Larg-A [184]; Lebectin [185]; Lebestatin [186]; LMW polysaccharide extracts from Agaricus blazei [187]; Lonicera japonica [188]; LMW undersulfated glycol-split heparin [142]; Low sulphated oligosaccharides from heparan sulphate [189]; Lysozime [190]
Marine-derived oligosaccharide sulfate [192]; Metastatin [193]; 2-Methoxyestradiol [194]; Methylene blue [195]; Methyltransferase inhibitors [163]; Microrganism fermantation [196]; Midkine [197]; Mitoxantrone [198]; Mixture of ascorbic acid, lisine, proline and gree tea [199]; Motuporanines [201]; Multiple RTK inhibitors [202]; Mustard essential oil [203]; Myo-inositol trispyrophosphate [204]
Neomycin [205]; Neridronate [206]; Neuregulin-2 [207]; Neurokinin-B [208]; Nitric oxide [173,210]; Nitrotoluene sulfonate [211]; Nonpeptide topomimetics [212]; Notch 4 [213]; Nucleolin antagonist [214]; Obtustatin [215]
Octacosanol [216]; Oncothanin [217]; 5’-O-trityl nucleoside analogs [218]; Opioid peptides [219]; Oriental herbal [220]; Oxaliplatin [221]; Paclitaxel [104]; PAI-1 [222]; PAK1[105]; PE [223]; Pedicularioside G [224]; PGG [225]; Pentosan polysulfate [226]; Pentraxin 3 [227]; Peptide trivalent arsenical [228]; Perillyl alcohol [229]; PPAR agonists [230]; PEX [231]; Phenethyl isothiocyanate [232]; Phenolic compounds [233]; P-henylenabil selenocyanate [234]; Philinopside A [235]; Phorbol esters [236]; Photodynamic therapy [237]; Piperazine derivative [238,239]; Placental ribonuclease inhibitor [240]; Plasma hyaluronan binding protein [241]; Plasminogen related protein [243]; PF4 [244,245]; PARP inhibitor [247]; Poly-L-lisyne/heparin [248]; Polysulphated derivative of tlaminarin [183]; Pomegranate [249]; Prenylnaringenin [250]; Prolactin [150,246]; Proline analogs [251]; Protamine [252]; PAR-1 antagonists [253]; Prothrombin fragments and rh prothrombin kringles [254,266]; Pyrimidines [255]; Purine analogues [256]; Purine riboside [257]; Pyracoumarin compounds [258]; Pyrazine [259]; p38 MAPK [260]
Quinoline [262]; Radicicol [263]; RDG-peptidomimetic [264]; Rh plasminogen kringle 1-3 [265]; Recombinant kringle domains of plasminogen, tissue-type plasminogen activator and urokinase [267,268,269]; Red wine [134]; Resveratrol [17]; Retinoids [270]; Rhodostomin [271]; Ribavirin [272]; Ribonuclease inhibitor [273]; Rosiglitazone [274]; Ruthenium red [275]
Safrole oxide [276]; Salmosin [277]; Sangivamycin [278]; Sanguinarine [279]; Saurus chinensis [280]; Sedun sarmentosum [281]; Serpin [282]; Sesterterpenes [283]; PF-4 [284]; Short peptide[286]; Simvastatin [287]; SJ-8002 [288]; S-nitrosocaptopril [289]; Sodium caffeate [290]; Solanum nigrum [291]; Somatostatin [292]; Somocystinamide [293]; Soy isoflavones [294]; S-phosphonate [295]; Spironolactone [296]; Squalamine [297]; Staurosporine [298]; Sulphated GAGs[300]; Sulfated polysaccharide-peptidoglican [301,302]; Sulfonated derivative of dystamycin [303,304]; Sulfonic acid polymers [305]; Sulf-2[306]; Sulindac analogue [307]; Sulindac [308]; Suramin [309,310,311]; Synthetic Grb2-Src [312]; Synthetic inhibitor of arylsulfatase [211]
Taraxacum officinale [314]; Taspine [315]; TAU 1120 [316]; Taxol [317]; Temozolomide [318]; Tenasum-C [319]; Terbinafine [320]; Terpenoids [321]; Tetrac [322]; Tetrameric tripeptide [323]; TGF-b[324]; Thalidomide metabolites [153]; 6-Thioguanine [325]; TSP-1 [326,327]; TP inhibitors [327]; Thymosin peptides [328]; Tinzaparin [329]; TIMP-3 [330]; TFPI [331]; Titanocene dichloride [332]; TNP470+ IFNa [333]; Tocotrienol [334]; Tocotrinol [335]; Topoisomerase inhibitors [336,337]; Topotecan [338]; Torilin [339]; Trapidil [340]; Triamcinolone acetonide [341]; Tricyclodecan-9-yl-xanthate [162]; Triphenylmethanem [50]; Tripterygium wilfordii [342]; Triptolide [343]; Triterpene acids [344]; Trypanosoma cruzi calreticulin [345]; Tyrosine phosphatase inhibitor [346]; TZT-1027 [347]
Ulmus davidiana var.japonica [348]; Undersulfated, LMW glycol-split heparin [349]; Ursodeoxycholic acid [350]; Ursolic and oleanolic acid [344]; Valproic acid [351]; Vanillyl alcohol [352]; VEGI [353]; VASH1B [354]; Vasostatin [245,355]; VEGF-toxin conjugate [356]; Vinblastine+rapamycic [357,358]; Vitamin D binding protein [359]; Vitamin D3 analogues [360]; Vitreous [361]; von Hippel-Lindau protein [362]; Wogonin [363]; Zoledronic acid [364]

* References between brackets.

## 3. Disadvantages of the CAM Assay

The major disadvantage of CAM is that it already contains a well-developed vascular network and the vasodilation that invariably follows its manipulation may be hard to distinguish from the effects of the test substance. Another limitation in nonspecific inflammatory reaction from the implant. Histologic study of CAM sections demonstrates the presence of perivascular inflammatory infiltrate together with any hyperplastic reaction of the chorionic epithelium. Nonspecific inflammatory reactions are much less frequent when the implant is made very early in CAM development and the host’s immune system is relatively immature [6]. Moreover, it might emphasize that species-specific differences might arise if one attempt to test the effects of high affinity antibodies generated against human surface antigens. However, to circumvent this drawback it is useful to perform the experiments early in the CAM development, since at that time the host’s immune system is relatively immature [6].

## 4. Concluding Remarks

CAM is widely utilized as an *in vivo* system to study antiangiogenesis. It offers the advantage of being relatively inexpensive and lends itself to large-scale screening, by using various stimulators alone or in combination with an antiangiogenic agent to examine the effectiveness of an inhibitor. The principal restrictions to its use are essentially due to nonspecific inflammatory reactions and to the presence of pre-existing vessels which make it difficult to determine the true extent of antiangiogenesis.
